# Supplementary material for: Unraveling metal effects on CO2 uptake in pyrene-based metal-organic frameworks
Source: Nat Commun. 2025 Feb 11;16:1516. doi: 10.1038/s41467-025-56296-w (PMC11814143; doi:10.1038/s41467-025-56296-w)
Supplement: Supplementary file 1 — Supplementary Information [file 41467_2025_56296_MOESM1_ESM.pdf]

Supporting Information: Unraveling Metal Effects on CO_2 Uptake in Pyrene-based Metal-Organic Frameworks

Nency P. Domingues,† Miriam J. Pougin,† Yutao Li,† Elias Moubarak,† Xin Jin,† F. Pelin Uran,† Andres Ortega-Guerrero,†,‡ Christopher P. Ireland,† Pascal Schouwink,¶ Christian Schürmann,§ Jordi Espín,|| Emad Oveisi,⊥ Fatmah Mish Ebrahim,†,# Wendy Lee Queen,|| and Berend Smit*,†

†*Laboratory of Molecular Simulation (LSMO), Institut des Sciences et Ingénierie Chimiques, École Polytechnique Fédérale de Lausanne (EPFL), Rue de l'Industrie 17, 1951 Sion, Switzerland.*

‡*Nanotech@surfaces Laboratory, Empa - Swiss Federal Laboratories for Materials Science and Technology, 8600 Dübendorf, Switzerland*

¶ *X-ray Diffraction and Surface Analytics Platform, École Polytechnique Fédérale de Lausanne (EPFL), Rue de l'Industrie 17, 1951 Sion, Switzerland.*

§ *Rigaku Europe SE, Hugenottenallee 167, 63263 Neu-Isenburg, Germany*

|| *Laboratory for Functional Inorganic Materials (LFIM), Institut des Sciences et Ingénierie Chimiques, École Polytechnique Fédérale de Lausanne (EPFL), Rue de l'Industrie 17, 1951 Sion, Switzerland.*

⊥*Interdisciplinary Centre for Electron Microscopy (CIME), École Polytechnique Fédérale de Lausanne (EPFL), 1015 Lausanne, Switzerland*

Cavendish Laboratory, School of Physical Sciences, University of Cambridge, Cambridge, United Kingdom.

E-mail: berend.smit@epfl.ch

Contents

1 Synthesis of the Pyrene Ligand (TBAPy) S-4

2 Computational Models S-6

3 Rietveld Refinements S-8

4 Ga-TBAPy Synthesis S-9

5 Crystal Structure Determination of Ga-TBAPy S-9

6 Ga-TBAPy Synchrotron *In-situ* Variable PXRD S-10

7 Brunauer–Emmett–Teller (BET) & Pore Volume Analysis S-11

8 Thermal Gravimetric Analysis (TGA) S-12

9 Scanning Electron Microscope (SEM) Images S-13

10 Physical Parameters for Uptake Normalization S-14

11 CO_2 Adsorption Isotherms S-15

12 Structural Flexibility S-16

13 N_2 Adsorption Isotherms S-17

14 DFT Binding Energies S-18

15 Heat Of Adsorption S-18

16 Density Maps S-21

17 Al_xSc_y-TBAPy PXRD Analysis S-22

18 Al_xSc_y-TBAPy SEM Images & Energy Dispersive X-Ray Analysis (EDX) S-23

19 Al_xSc_y-TBAPy Data S-24

References S-26

1 Synthesis of the Pyrene Ligand (TBAPy)

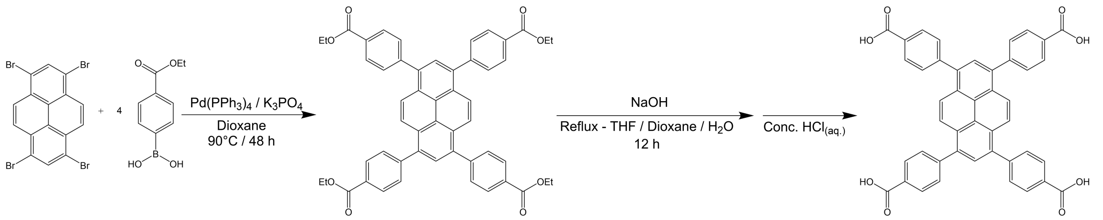

Figure S1: Two step synthesis of 1,3,6,8-tetrakis($p-$benzoicacid)pyrene (TBAPy).

Synthesis of 1. Procedure adapted from Wang et al.[S1]. Dioxane (270 mL) was added into a 500 mL three-necked round-bottom flask equipped with a magnetic stirrer and water condenser. Dioxane was put under constant stirring and degassed with N_2 for approximately 1.5 hours. With the N_2 still purging and the dioxane under stirring, 1,3,6,8-tetrabromopyrene (5 g), 4-(ethoxycarbonylphenyl)boronic acid (8.25 g), potassium phosphate tribasic (16.5 g) and tetrakis(triphenylphosphine)-palladium(0) (0.75 g) were inserted into the flask (the solution turned brown). The opening of the round-bottom flask was plugged with a glass pug, and the system was purged with N_2 for an additional 5 minutes. With the help of a heating mantle, the suspension was heated to 90 °C for 48 h to 72 h. The color of the suspended solid should become more yellow as the reaction proceeds. The solution turned black at the end of the reaction. Once the reaction was complete, water (200 mL) was added to the reaction mixture, and the mixture was let to cool down. The reaction mixture was then filtered with a glass Büchner funnel (200 mL), with a medium frit. The yellow solid was collected on the frit and washed with water (2x 100 mL) and acetone (200 mL). Boiling chloroform (300 mL) was then poured onto the glass frit to dissolve the desired product. Methanol (300 mL) was added to the solution which was at room temperature. A light yellow precipitate was formed as methanol was added to the solution. The suspension was let to sit for 30 minutes and the yellow solid was collected with a glass Büchner funnel (200 mL) with a medium filter. The product was

dried overnight in a vacuum oven at 70 °C. Approximately 4 g of 1 (i.e., 1,3,6,8-tetrakis(4-(methoxycarbonyl)phenyl)pyrene) should be obtained. The product was analyzed by ^{1}H-NMR spectroscopy by dissolving it in Chloroform-d.

Synthesis of 1′. Procedure adapted from Stylianou et al.[S2]. Product 1 (1 g) was dispersed in a solvent mixture THF/dioxane/H_2O (ratio 5/2/2) (100 mL). Concentrated NaOH (20 mL) was added to it. The mixture was then stirred under reflux at 85 °C overnight. Once the reaction was complete, water was added to the suspension, and a clear yellow solution was formed. The solution was stirred at room temperature for 1.5 hours. Using concentrated HCl (32 wt%), the pH of the solution was adjusted to 2. A yellow precipitate was formed, collected by filtration, and washed with water, HCl (1 M), and diethyl ether. The solid was then dried under vacuum in a ventilated oven at 70 °C overnight. Once the product was properly dry, boiling DMF was added to the yellow powder. The solution was left under stirring and filtered before cooling to room temperature. Once the solution had cooled down, dichloromethane (300 mL) was added, and a yellow solid was formed. The mixture was filtered with a glass Büchner funnel (200 mL) with a medium filter, and the powder was dried in a vacuum oven at 70 °C overnight to obtain 1′ (TBAPy) (0.78 g). The product was analyzed by ^{1}H-NMR spectroscopy by dissolving it in DMSO-d_6.

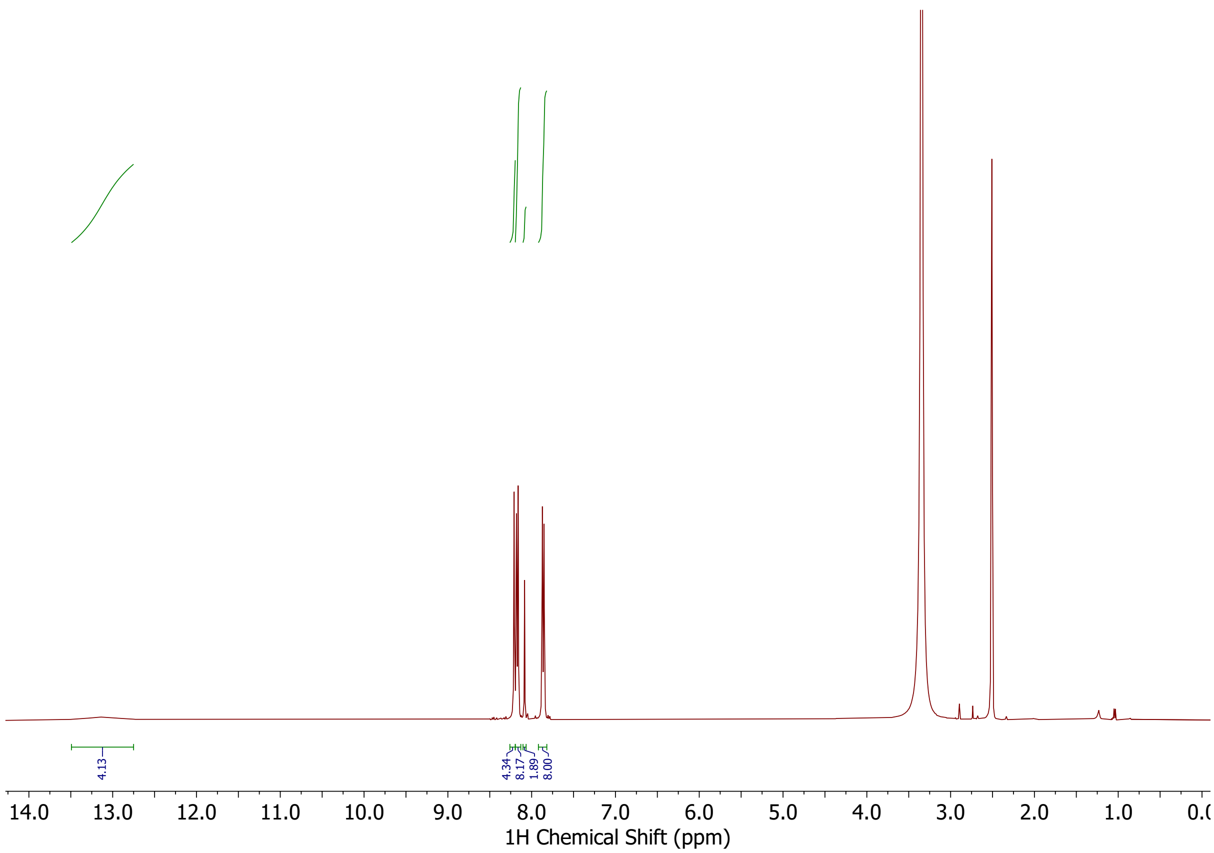

Figure S2: ^{1}H-NMR (DMSO-d_6) of 1′ (i.e., 1,3,6,8-tetrakis(p−benzoicacid)pyrene (TBAPy): δ 7.86 (d, J = 8.4 Hz, 8H), 8.08 (s, 2H), 8.17 (d, J = 8.4 Hz, 8H), 8.21 (s, 4H), 13.1 (s, 4H). Source data are provided as a Source Data file.

2 Computational Models

Orthorhombic M-TBAPy (with M = Al, Ga, In, and Sc). The computational models of M-TBAPy were derived using the reported coordination file for orthorhombic Al-TBAPy,[S3] swapping Al for Ga, In, and Sc, respectively. The cell parameters were fully relaxed with the cell optimization simulation in DFT, except for the reported Al-TBAPy.

Monoclinic M-TBAPy (with M = Ga, and In). The monoclinic models were built based on the crystallographic information of the micro-electron diffraction (microED/3D-ED) of the Ga-TBAPy structure (see next paragraph). For *m*-Ga-TBAPy, the cell parameters

were kept unchanged and only the geometry coordinates were optimized in DFT. For *m*-In-TBAPy, we replaced Ga with In, and the cell parameters were fully relaxed with DFT. We also performed DFT geometry optimization for the monoclinic structure of In-TBAPy.

Orthorhombic $Al_{0.50}Sc_{0.50}$-TBAPy. The computational model of $Al_{0.50}Sc_{0.50}$-TBAPy was constructed based on the crystallographic data of orthorhombic Al-TBAPy. A mixed-metal framework was created by replacing half of the Al atoms with Sc atoms. The cell parameters were fully relaxed with the cell optimization simulation in DFT.

3 Rietveld Refinements

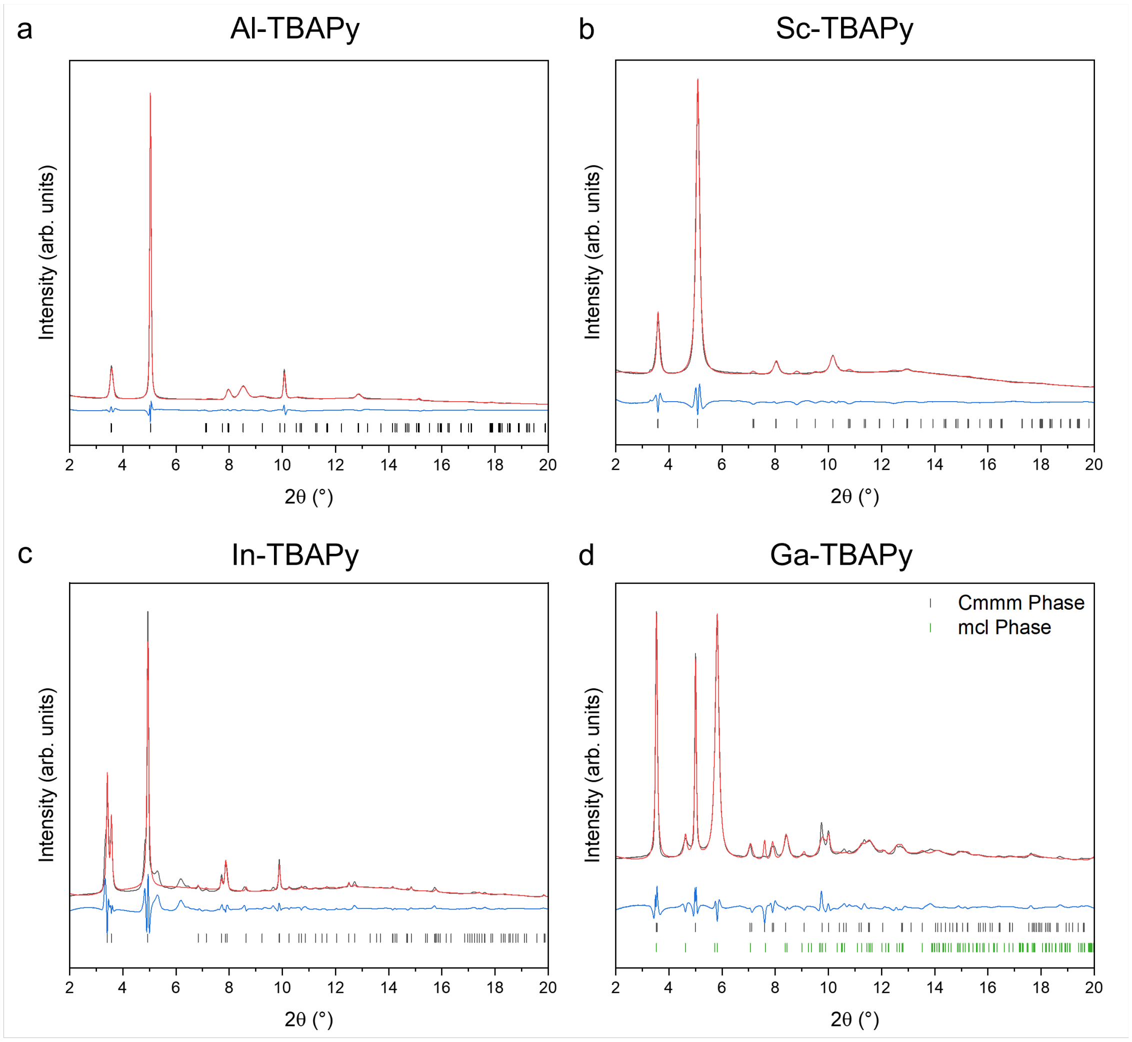

Figure S3: Rietveld refinements of the activated **a)** Al-TBAPy, **b)** Sc-TBAPy, **c)** In-TBAPy, and **d)** Ga-TBAPy with λ = 0.9572 Å. Color code: observed diffraction pattern (black), calculated (red), difference (blue). The *hkl* phase tags correspond to the main *Cmmm* phase of Al-, Sc- and In-TBAPy, while for Ga-TBAPy the *hkl* phase tags for the *mcl* phase are also shown. Source data are provided as a Source Data file.

4 Ga-TBAPy Synthesis

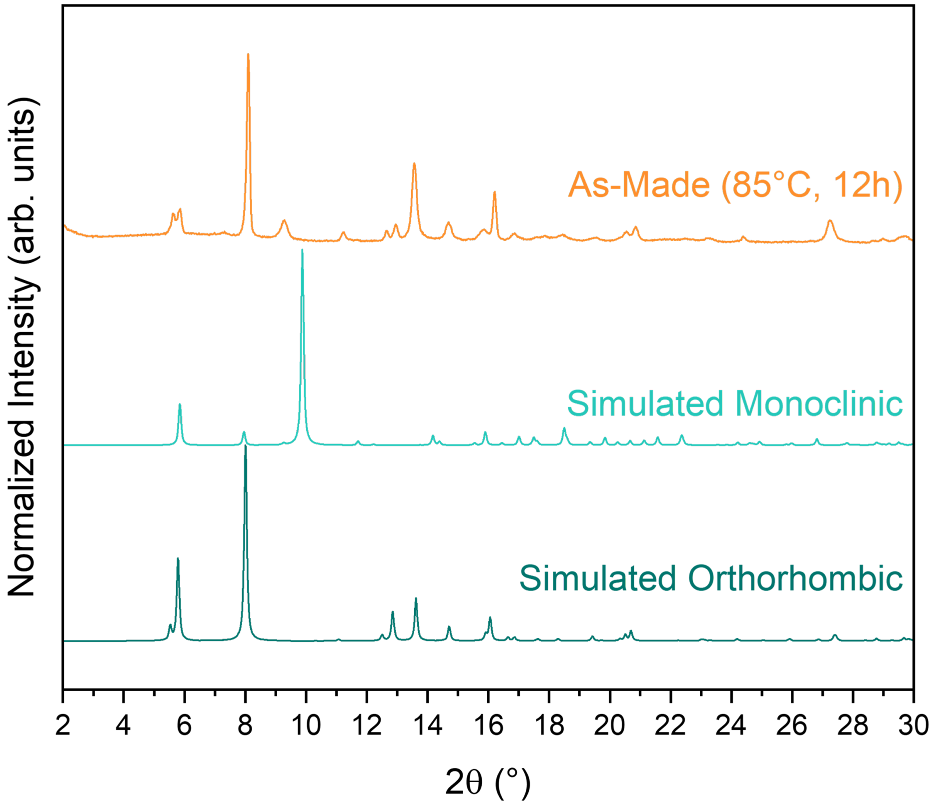

Figure S4: PXRD patterns of the simulated orthorhombic and monoclinic Ga-TBAPy, as well as the as-made material synthesized at 85 °C for 12h, showcasing the characteristic Bragg diffraction peaks of the monoclinic and orthorhombic phases present in the sample. Source data are provided as a Source Data file.

5 Crystal Structure Determination of Ga-TBAPy

The sample consisted of small, flake-like crystallites of ca 1 μm widths and a thickness of ca 0.1 μm. The data of the single measurements were incomplete with completeness below 70%. For the final refinement, data from three crystallites were merged. While each individual data set was suitable to provide a correct structure solution, by merging multiple data sets, more complete, significant, and redundant data were created and

resulted in a high-quality structure refinement. The selected data sets were collected in continuous rotation at a virtual detector distance of 650 mm in a tilt range of -40° to +70°, -60° to +60° and -70° to +70°, a step size of 0.25° and a scan speed of 1°/s at spot size 4 (of 5) with 10 μm CL and 100 μm SA apertures. The merged measurements resulted in a total experiment time of 13 minutes and a completeness of 96% for the structure in space group $P2/c$ for a resolution of up to 0.84Å. Crystal Data for $C_{22}H_{12}GaO_5$ (M = 426.056 g/mol): monoclinic, space group $P2/c$ (no. 13), a = 11.1(4) Å, b = 15.1(3) Å, c = 12.3(4) Å, β = 90.12(7)°, V = 2052(106) Å^3, Z = 4, T = 298 K, μ(electrons) = 0.000 mm^{-1}, D_{calc} = 1.379 g/cm^3, 26518 reflections measured (0.2° $\leq 2\Theta \leq$ 1.8°), 3614 unique (R_{int} = 0.2644, R_{sigma} = 0.1629) which were used in all calculations. The final R_1 was 0.2425 (I>=2u(I)) and wR_2 was 0.5356 (all data). CCDC 2327953.

6 Ga-TBAPy Synchrotron *In-situ* Variable PXRD

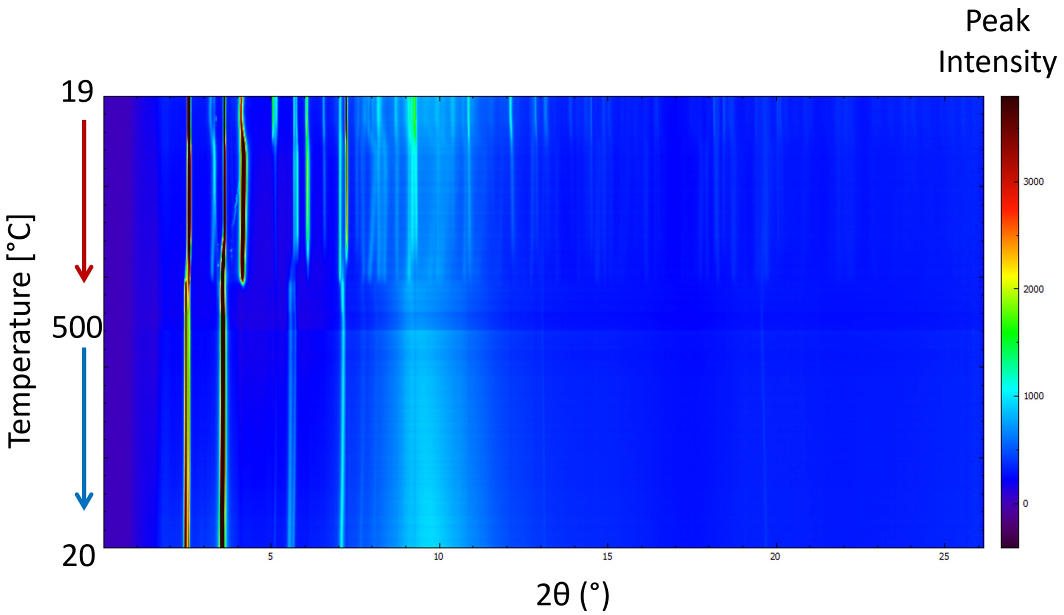

Figure S5: *In-situ* variable powder X-ray diffraction measurement of Ga-TBPAy with λ = 0.69437 Å. Source data are provided as a Source Data file.

7 Brunauer–Emmett–Teller (BET) & Pore Volume Analysis

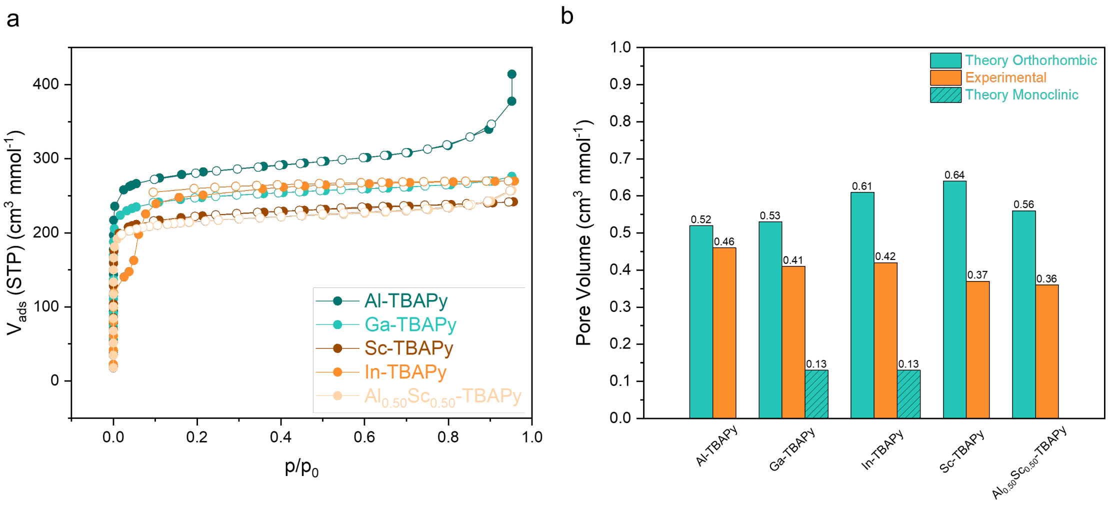

Figure S6: **a)** Experimental N_2 isotherm at 77 K; **b)** comparison of the experimental and simulated pore volumes for the M-TBAPy MOFs. The experimental pore volumes are determined from the N_2 isotherms at 77 K (for Al-TBAPy, the calculations were performed at $P/P_0 = 0.5$ to exclude the effects of condensation observed in the isotherms, while for the remaining MOFs, they were conducted at $P/P_0 = 0.8$. Source data are provided as a Source Data file.

8 Thermal Gravimetric Analysis (TGA)

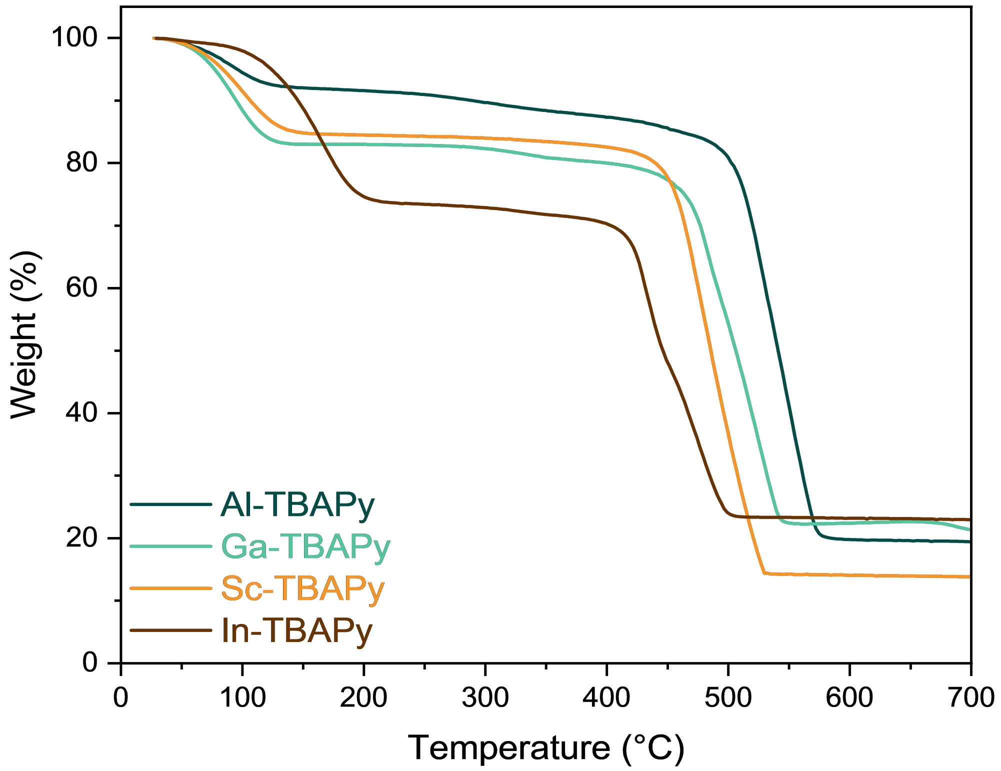

Figure S7: Thermal gravimetric analysis (TGA) of the M-TBAPy MOFs: weight (%) as a function of temperature (°C). Source data are provided as a Source Data file.

9 Scanning Electron Microscope (SEM) Images

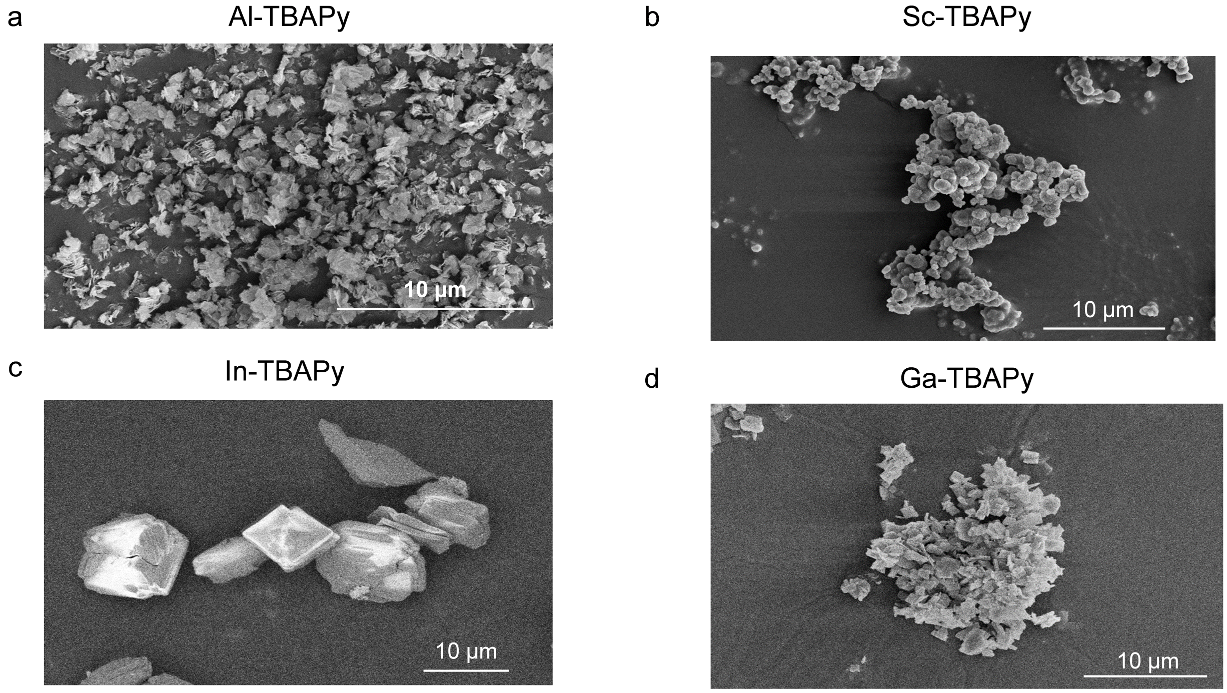

Figure S8: Scanning-electron microscope (SEM) images of the as-made materials of: **a)** Al-TBAPy, **b)** Sc-TBAPy, **c)** In-TBAPy, and **d)** Ga-TBAPy.

10 Physical Parameters for Uptake Normalization

Table S1: Computational physical parameters obtained for the optimized CIFs considering $M_2(OH)_2$(TBAPy). The differences in density and cell volume values for the In- and Ga-TBAPy structures for the different orthorhombic and monoclinic configurations explain the use of the molecular weight for normalization purposes. The density and cell volume of $Al_{0.50}Sc_{0.50}$-TBAPy are not provided given the slight variations due to the CIF considered as all structures are cell optimized individually, while their molecular weight is uniform across the different CIFs.

MOF	Unit Cell	Density (g cm^{-3})	Cell Volume (Å^3)	Molecular Weight (g mmol^{-1})
**Al-TBAPy**	**Orthorhombic**	0.800	1591	0.767
**Sc-TBAPy**	**Orthorhombic**	0.744	1791	0.803
**In-TBAPy**	**Orthorhombic**	0.897	1744	0.943
	**Monoclinic**	1.450	1079	0.943
**Ga-TBAPy**	**Orthorhombic**	0.874	1620	0.852
	**Monoclinic**	1.373	1031	0.852
**$Al_{0.50}Sc_{0.50}$-TBAPy**	**Orthorhombic**	-	-	0.785

11 CO_2 Adsorption Isotherms

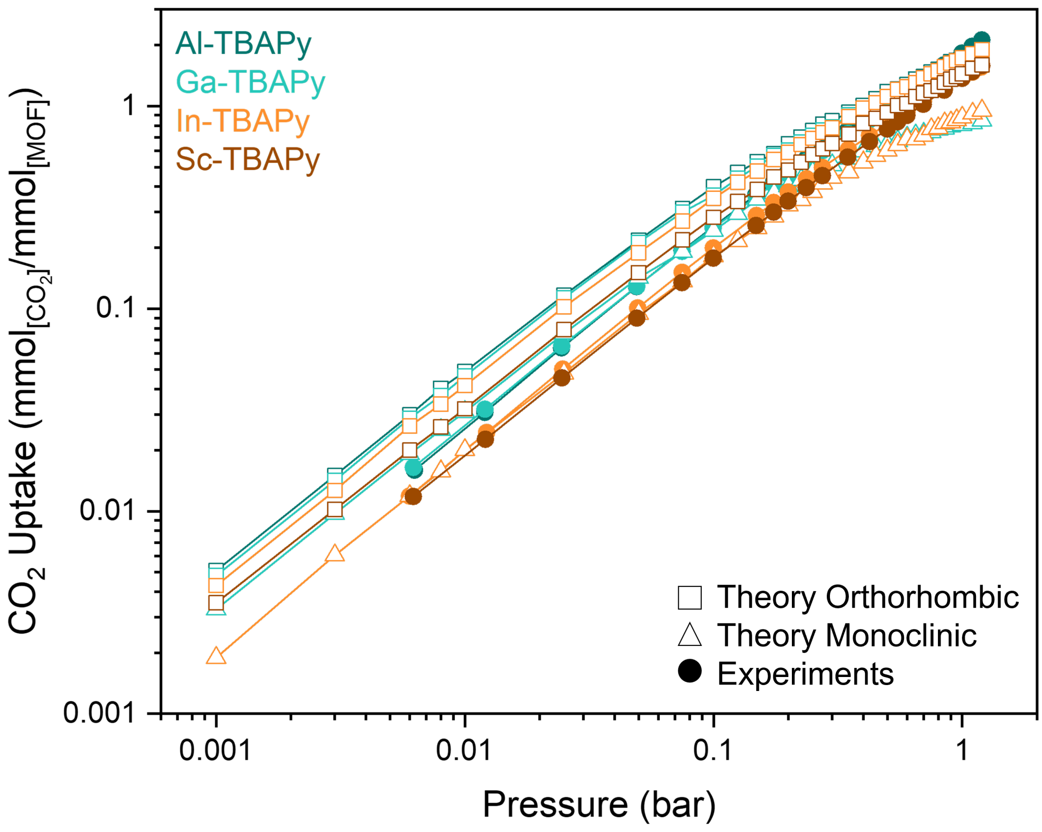

Figure S9: CO_2 adsorption isotherms of the M-TBAPy MOFs in $\mathrm{mmol}_{[CO_2]}/\mathrm{mmol}_{[MOF]}$ on a log-log scale. Experimental isotherms at 40 °C (filled circles). Predicted isotherms of the orthorhombic structures (empty squares), predicted isotherms of the monoclinic structures (empty triangles).

12 Structural Flexibility

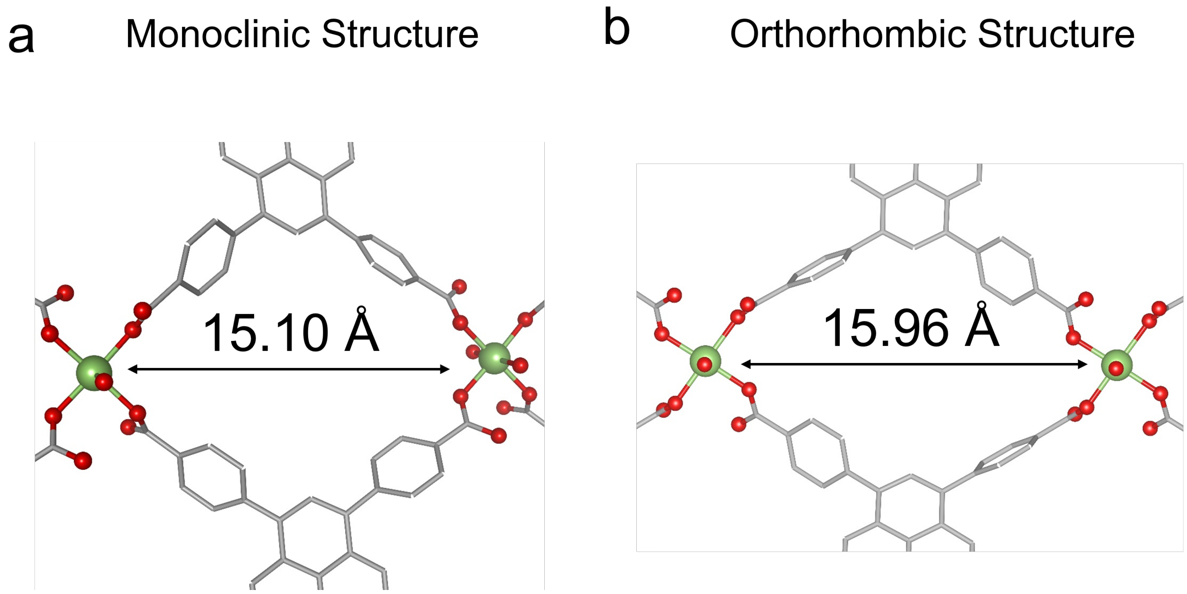

Figure S10: Zoomed-in image of pore C for the **a)** monoclinic and **b)** orthorhombic structures of Ga-TBAPy, showcasing the stretching of the pore. Color code: C (grey), O (red), In or Ga (light green).

13 N_2 Adsorption Isotherms

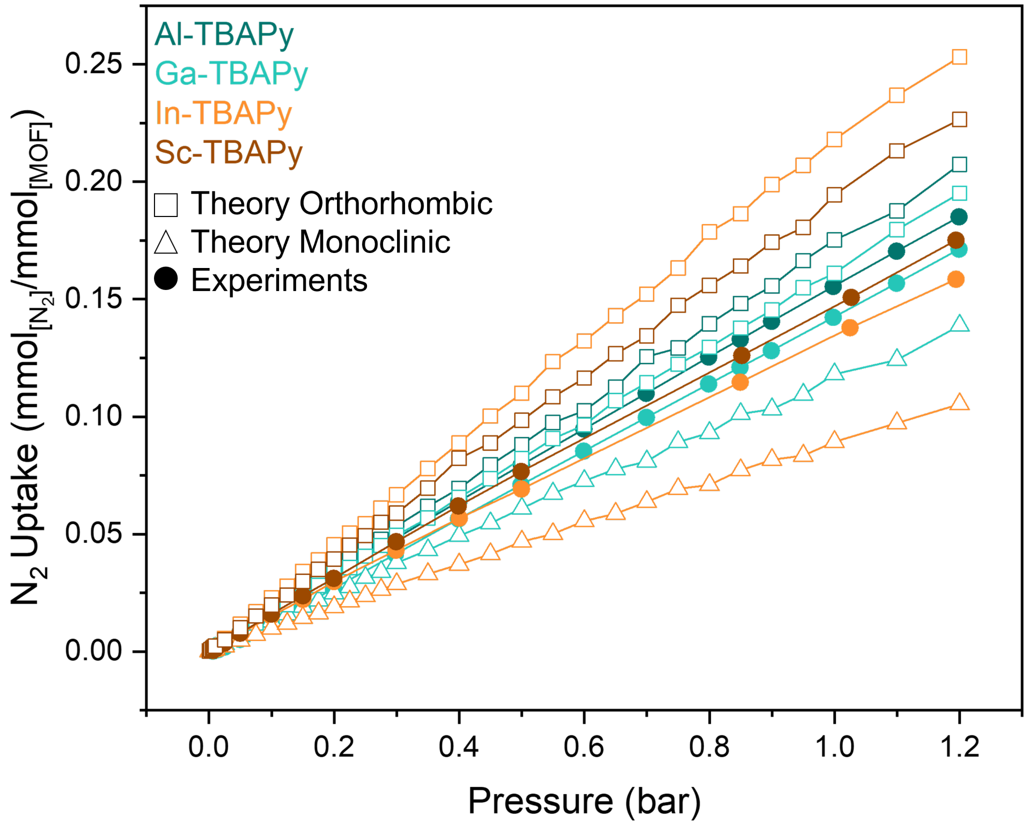

Figure S11: N_2 adsorption isotherms of the M-TBAPy MOFs in $mmol_{[CO_2]}/mmol_{[MOF]}$ on a linear scale. Experimental isotherms at 40 °C (filled circles). Predicted isotherms of the orthorhombic structures (empty squares), predicted isotherms of the monoclinic structures (empty triangles). Source data are provided as a Source Data file.

14 DFT Binding Energies

Table S2: Computed binding site energies obtained from DFT calculations for CO_2, N_2 and H_2O molecules for all MOFs in their respective configurations. Interaction energies in the minimum energy configuration from DFT calculations (i.e., $E_{CO_2,min}$) are computed using the binding-site workflow. For the orthorhombic frameworks, the CO_2-MOF interactions in the different channels are tabulated as $E_{CO_2,PoreB}$ and $E_{CO_2,PoreC}$. For H_2O, site A refers to the site in between the two pyrene ligands, and site B refers to the binding site closer to the metal rod.

MOF	Crystal System	DFT Binding Energies (kJ/mol)					
		$E_{CO_2,min}$	$E_{CO_2,PoreB}$	$E_{CO_2,PoreC}$	E_{N_2}	$E_{H_2O,SiteA}$	$E_{H_2O,SiteB}$
**Al-TBAPy**	Orthorhombic	-32.0	-21.9	-21.0	-25.0	-24.1	-41.0
**Ga-TBAPy**	Orthorhombic	-27.5	-23.5	-23.2	-25.5	-26.1	-32.2
	Monoclinic	-30.9	-	-	-22.0	0	-46.4
**In-TBAPy**	Orthorhombic	-29.1	-22.6	-22.2	-25.8	-25.5	-40.0
	Monoclinic	-27.7	-	-	-18.7	0	-41.4
**Sc-TBAPy**	Orthorhombic	-27.2	-25.2	-23.6	-21.4	-22.0	-42.7

15 Heat Of Adsorption

Experimental CO_2 adsorption isotherms were collected at 25, 40, and 55 °C and fitted to a dual-site Langmuir model following:

$$q = q_{sat,1}\frac{b_1 P}{1 + b_1 P} + q_{sat,2}\frac{b_2 P}{1 + b_2 P} \tag{1}$$

where q corresponds to the adsorbed amount in mmol/g, $q_{sat,1}$ is the adsorption capacity for site 1, b_1 is the Langmuir parameter for site 1 ($q_{sat,2}$ and b_2 are equivalent for site 2) and P is the pressure in Pa.

Subsequently, the Clausius-Clapeyron equation (2) was used to calculate the isosteric enthalpy of adsorption (i.e., Q_{st}) for CO_2:

$$\ln(P) = -\frac{Q_{st}}{R}\left(\frac{1}{T}\right) + c \tag{2}$$

where R is the ideal gas law constant, T is the temperature, and c is a constant.

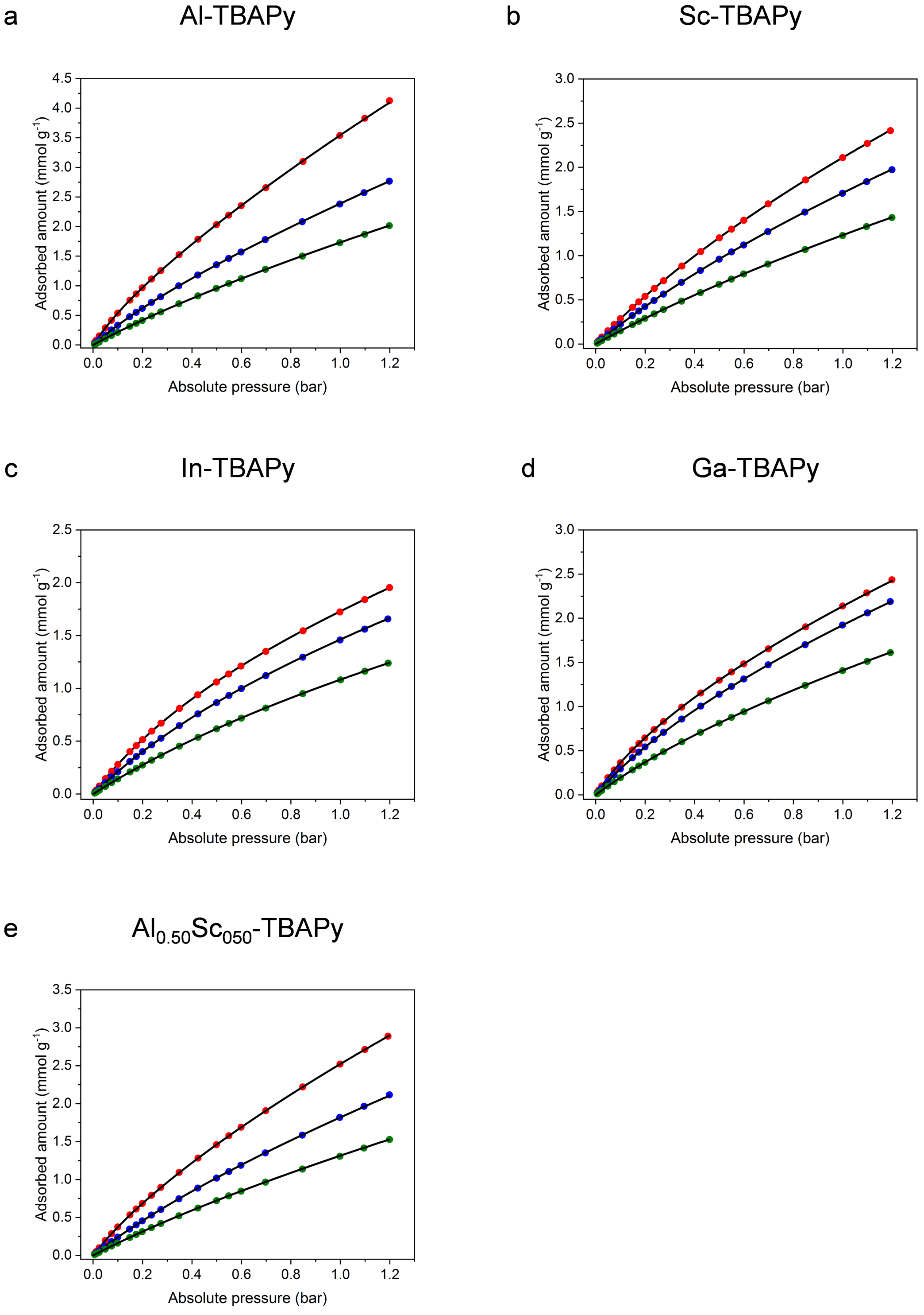

Figure S12: Variable temperature CO_2 isotherms at 25 °C (red), 40 °C (blue) and 55 °C (green) for **a)** Al-TBAPy, **b)** Sc-TBAPy, **c)** In-TBAPy, **d)** Ga-TBAPy, **e)** $Al_0 \cdot 50\,Sc_0 \cdot 50$-TBAPy. Source data are provided as a Source Data file.

Table S3: Langmuir parameters for all MOFs studied in this work.

MOF	Temperature (°C)	$q_{sat,1}$	b_1	$q_{sat,2}$	b_2
	25	34.5	9.4466E-07	0.69	4.5597E-05
**Al-TBAPy**	40	34.5	5.8361E-07	0.69	2.3925E-05
	55	34.5	4.054E-07	0.69	1.2874E-05
	25	15.8	1.1504E-06	0.8	1.5126E-05
**Sc-TBAPy**	40	15.8	8.8158E-07	0.8	1.1682E-05
	55	15.8	5.957E-07	0.8	7.5924E-06
	25	16.4	5.7177E-07	1.38	1.5816E-05
**In-TBAPy**	40	16.4	4.7589E-07	1.38	1.0913E-05
	55	16.4	3.2584E-07	1.38	6.971E-06
	25	12.0	1.5213E-06	0.71	3.5055E-05
**Ga-TBAPy**	40	12.0	1.3478E-06	0.71	2.342E-05
	55	12.0	9.0329E-07	0.71	1.4188E-05
	25	18.0	1.3068E-06	0.58	3.2084E-05
**$Al_{0.50}Sc_{0.50}$-TBAPy**	40	18.0	8.805E-07	0.58	1.6682E-05
	55	18.05	5.9562E-07	0.59	1.0504E-05

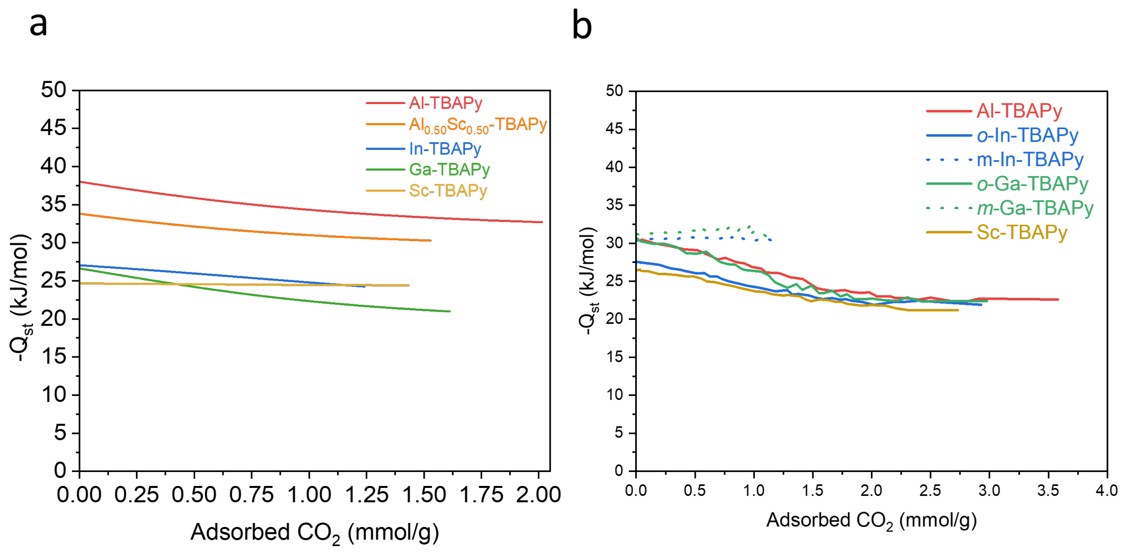

Figure S13: Experimental **a)** and **b)** computational (GCMC) heat of adsorption values, in kJ/mol, for all MOFs. Source data are provided as a Source Data file.

16 Density Maps

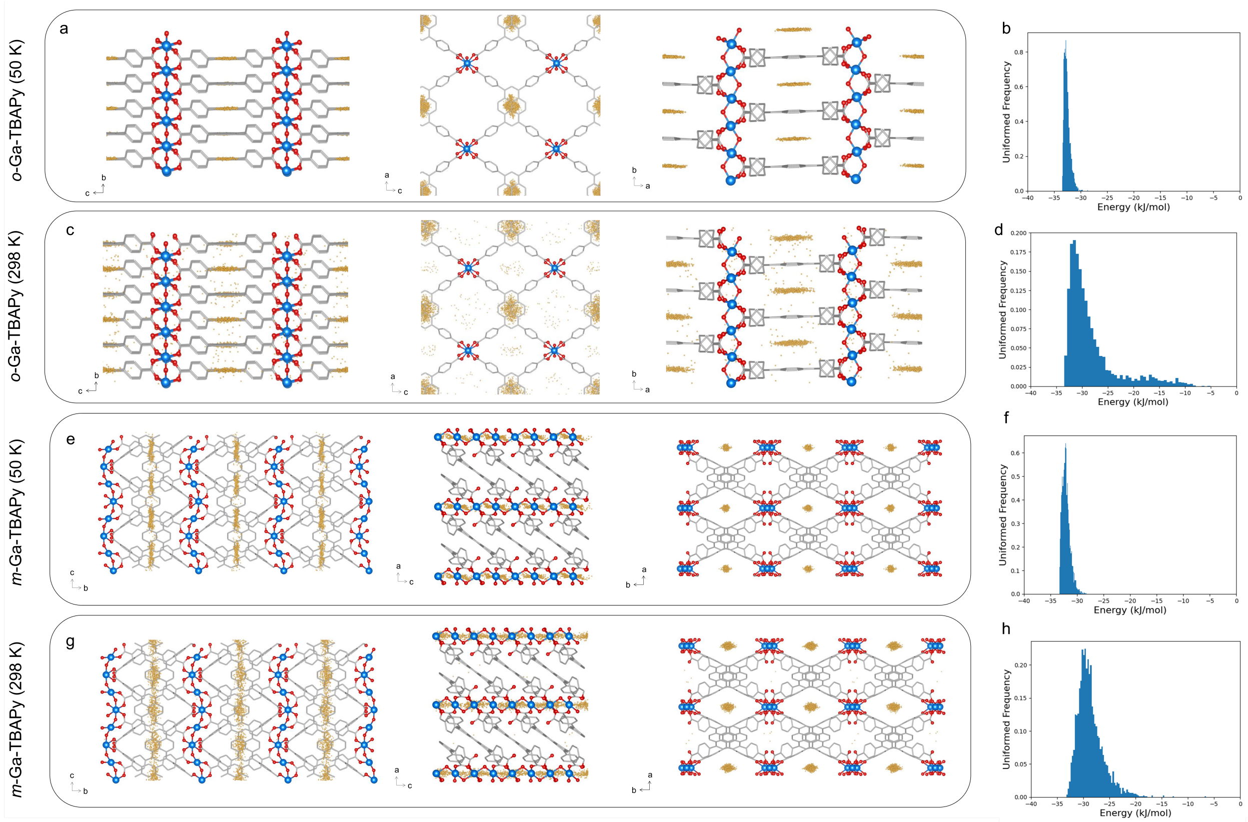

Figure S14: **a), c), e), and g)** CO_2 probability distribution for *o*-Ga-TBAPy at 50 K and 298 K, and *m*-Ga-TBAPy at 50 K and 298 K, respectively. **b), d), f), and h)** Histograms of the frequency as a function of the binding energy for those same structures and temperatures, respectively. Color code: C (grey), O (red), metal (light blue) CO_2 (yellow). Source data are provided as a Source Data file.

17 Al_xSc_y-TBAPy PXRD Analysis

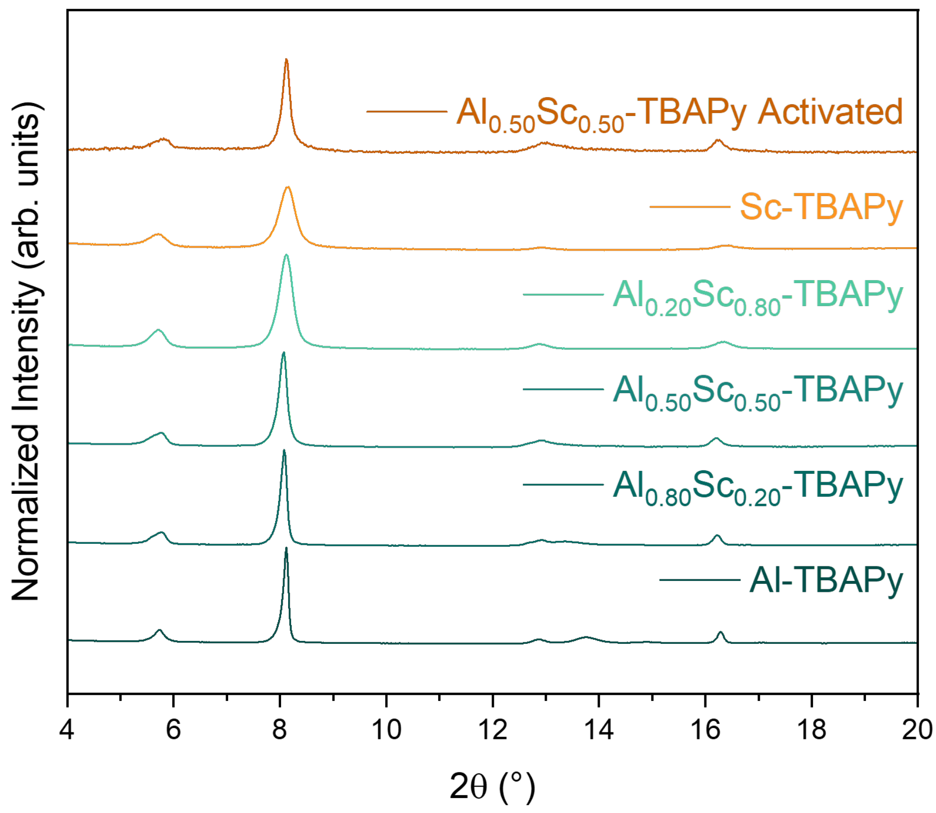

Figure S15: Measured PXRD of the pure Al- and Sc-TBAPy MOFs, as well as different Al_xSc_y-TBAPy mixtures with Al:Sc ratios of 20:80, 50:50, 80:20. The PXRD of $Al_{0.50}Sc_{0.50}$-TBAPy after activation at 170 °C, for 12 hours under vacuum is also shown. λ = 1.5406 . Source data are provided as a Source Data file.

Table S4: Lattice parameters obtained through Le Bail fits of transmission X-ray diffraction measurements, utilizing the *Cmmm* unit cell.

	a [Å]	*b* [Å]	*c* [Å]
**Al-TBAPy**	30.528	7.250	15.539
**$Al_{0.80}Sc_{0.20}$-TBAPy**	30.449	7.540	15.655
**$Al_{0.50}Sc_{0.50}$-TBAPy**	30.434	7.596	15.732
**$Al_{0.20}Sc_{0.80}$-TBAPy**	30.393	7.670	15.456
**Sc-TBAPy**	30.169	7.494	15.428

18 Al_xSc_y-TBAPy SEM Images & Energy Dispersive X-Ray Analysis (EDX)

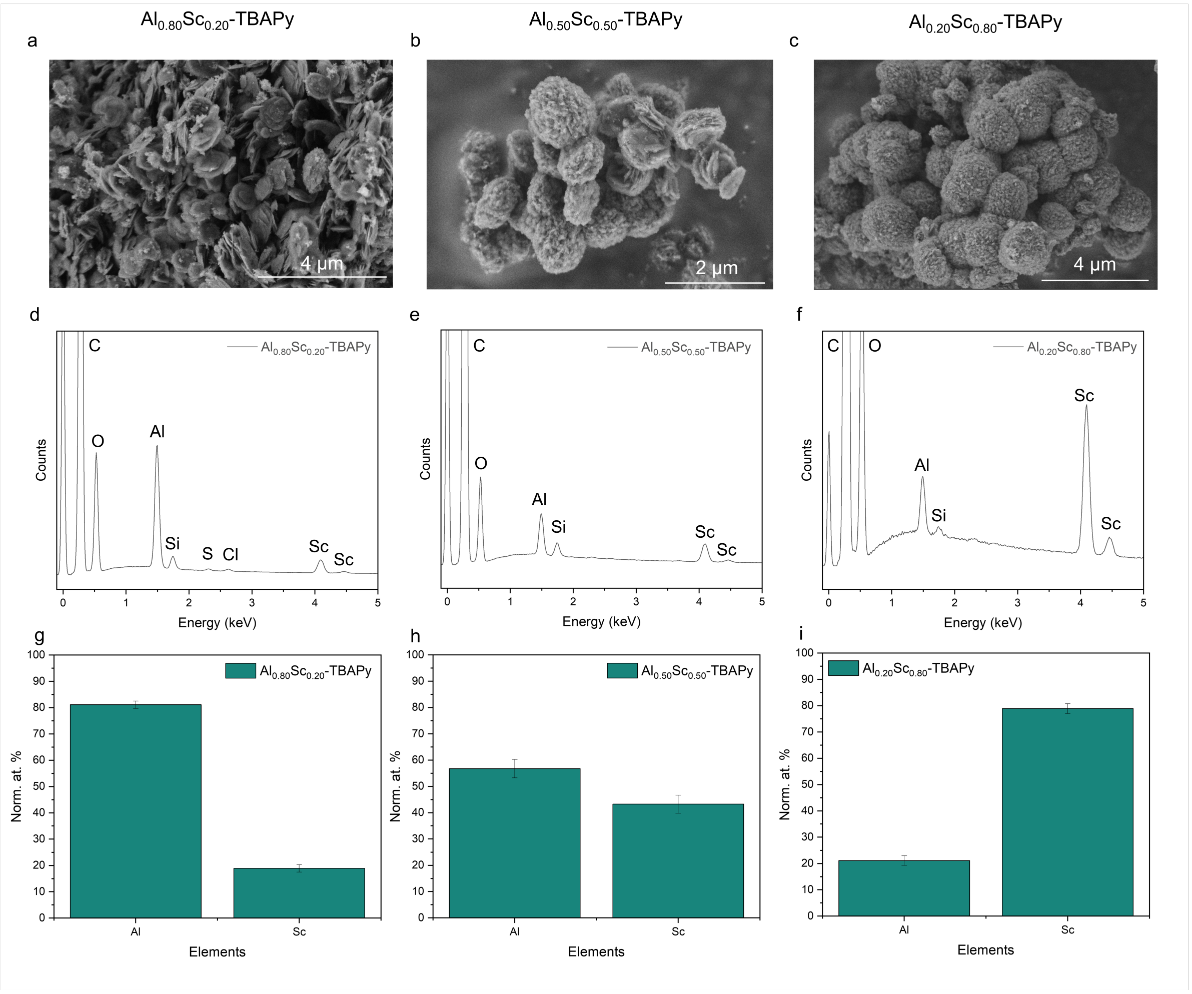

Figure S16: **a - c**) SEM images, **d - f**) EDX spectra and **g - i**) EDX quantified content of Al and Sc (in Norm. at. %) for $Al_{0.80}Sc_{0.20}$-TBAPy, $Al_{0.50}Sc_{0.50}$-TBAPy and $Al_{0.20}Sc_{0.80}$-TBAPy MOFs, respectively. Source data are provided as a Source Data file.

19 Al_xSc_y-TBAPy Data

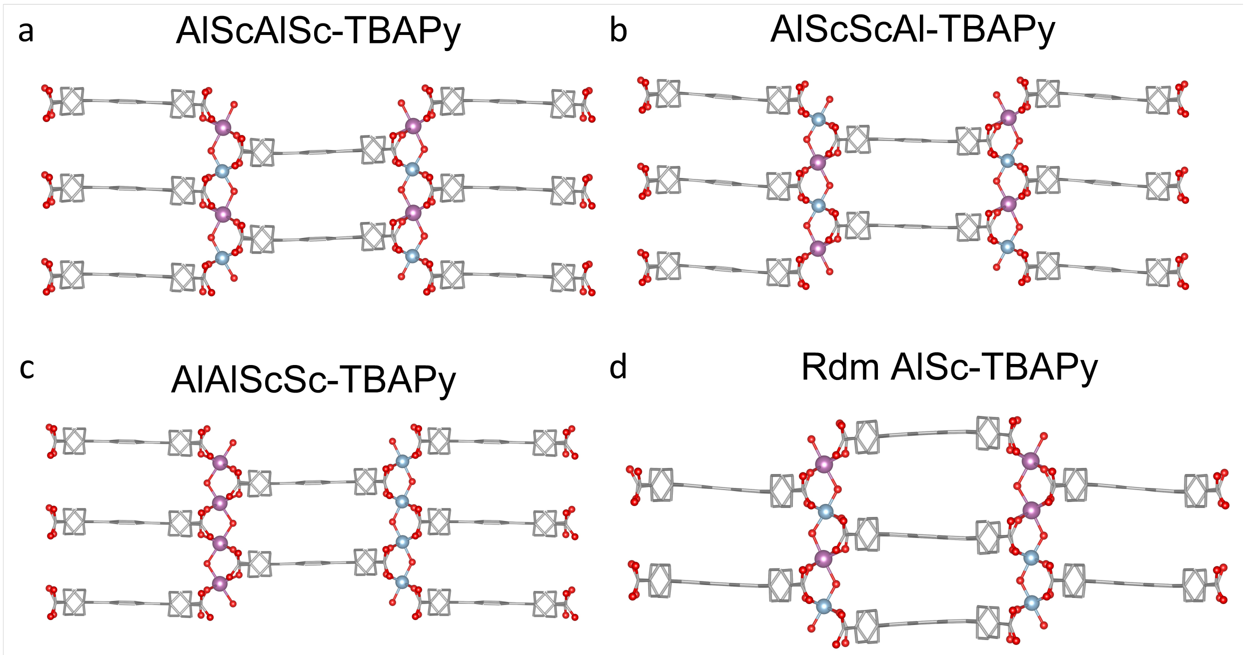

Figure S17: Crystal structure representations of the four different models developed for the $Al_{0.50}Sc_{0.50}$-TBAPy MOF. **a)** the structure alternates between Al and Sc ions with identical ions facing each other; **b)** depicts alternating Al and Sc ions with different ions facing each other; **c)** presents pure rods of Al ions and pure rods of Sc ions facing each other; **d)** shows a random distribution of Al and Sc ions throughout the structure. Color code: C (grey), O (red), Al (light blue), Sc (light pink). The ball-and-stick representation was used. Carbons (C) and hydrogens (H) were hidden for clarity.

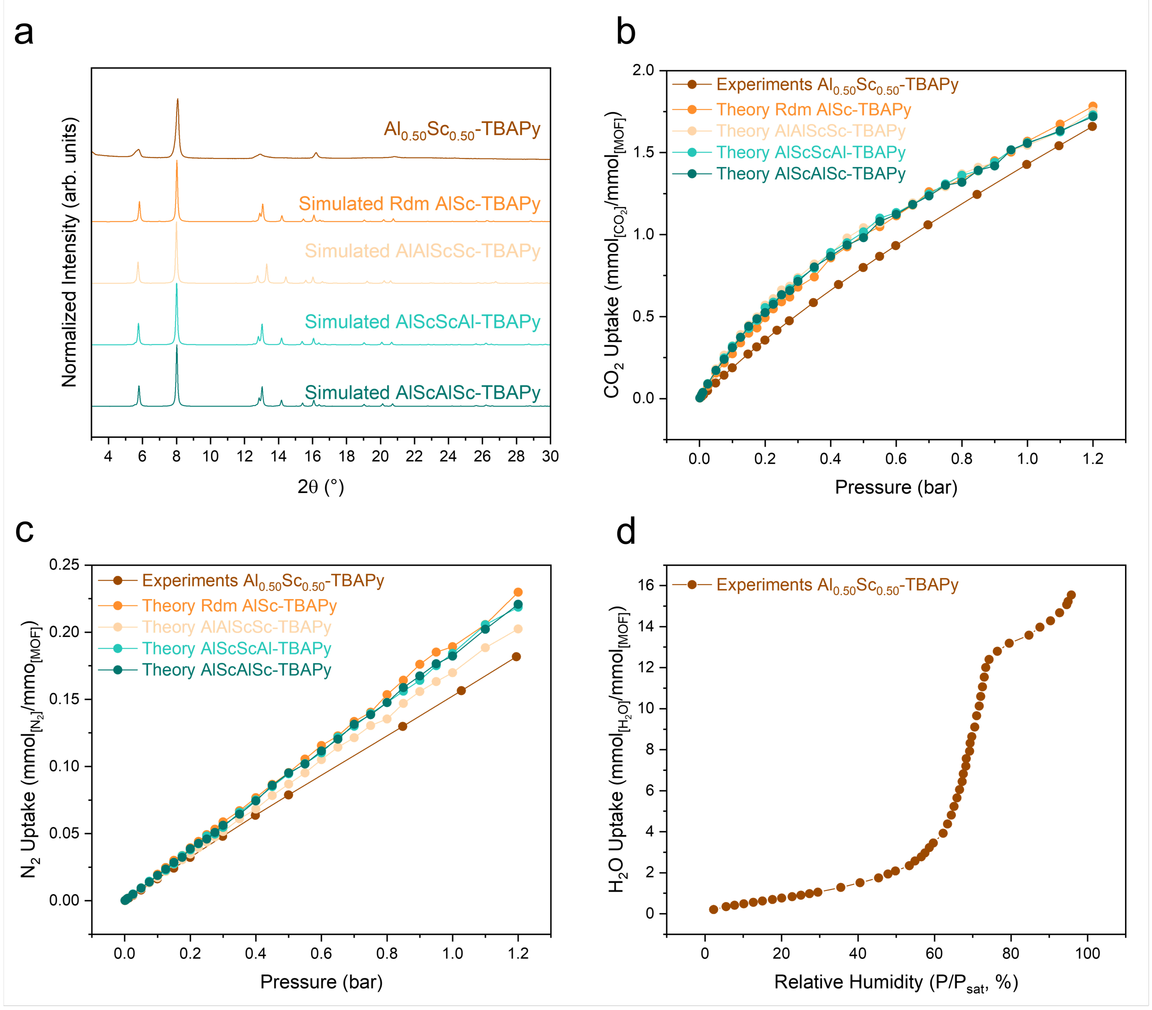

Figure S18: **a)** Experimental and simulated PXRD diffraction patterns; pure experimental and computational **b)** CO_2, and **c)** N_2, and experimental **d)** H_2O adsorption isotherms at 40 °C. Source data are provided as a Source Data file.

References

(S1) Wang, T. C.; Vermeulen, N. A.; Kim, I. S.; Martinson, A. B.; Stoddart, J. F.; Hupp, J. T.; Farha, O. K. Scalable synthesis and post-modification of a mesoporous metal-organic framework called NU-1000. *Nature protocols* **2016**, *11*, 149–162.

(S2) Stylianou, K. C.; Heck, R.; Chong, S. Y.; Bacsa, J.; Jones, J. T.; Khimyak, Y. Z.; Bradshaw, D.; Rosseinsky, M. J. A guest-responsive fluorescent 3D microporous metal-organic framework derived from a long-lifetime pyrene core. *Journal of the American Chemical Society* **2010**, *132*, 4119–4130.

(S3) Boyd, P. G.; Chidambaram, A.; García-Díez, E.; Ireland, C. P.; Daff, T. D.; Bounds, R.; Gładysiak, A.; Schouwink, P.; Moosavi, S. M.; Maroto-Valer, M. M., et al. Data-driven design of metal–organic frameworks for wet flue gas CO2 capture. *Nature* **2019**, *576*, 253–256.
